# Gene-Diet Interaction and Precision Nutrition in Obesity

**DOI:** 10.3390/ijms18040787

**Published:** 2017-04-07

**Authors:** Yoriko Heianza, Lu Qi

**Affiliations:** 1 Department of Epidemiology, School of Public Health and Tropical Medicine, Tulane University, New Orleans, LA 70112, USA; yheianza@tulane.edu; 2 Department of Nutrition, Harvard T.H. Chan School of Public Health, Boston, MA 02115, USA

**Keywords:** gene-diet interactions, weight loss, obesity

## Abstract

The rapid rise of obesity during the past decades has coincided with a profound shift of our living environment, including unhealthy dietary patterns, a sedentary lifestyle, and physical inactivity. Genetic predisposition to obesity may have interacted with such an obesogenic environment in determining the obesity epidemic. Growing studies have found that changes in adiposity and metabolic response to low-calorie weight loss diets might be modified by genetic variants related to obesity, metabolic status and preference to nutrients. This review summarized data from recent studies of gene-diet interactions, and discussed integration of research of metabolomics and gut microbiome, as well as potential application of the findings in precision nutrition.

## 1. Introduction

The increasing epidemic of obesity has coincided with a profound shift of our living environment, such as unhealthy dietary patterns, a sedentary lifestyle, physical inactivity, poor sleeping habits, as well as changes in demographic and cultural background [1]. In a recent study of the US national cohort, the magnitude of association between obesity, as assessed by body mass index (BMI) and genetic risk of obesity was stronger in more recent birth cohorts than in earlier years of birth cohorts, suggesting that such genetic predisposition to obesity may have a greater effect in more recent obesogenic environments [2]. Although the environmental risk factors are largely modifiable and the development of obesity would be essentially preventable, genetic variants associated with adiposity may also influence behavioral responses such as shaping appetite, total energy intake and preferences of macronutrients [3,4,5,6]. Also, food preference patterns (such as high sugar and carbohydrate consumption) [7,8] would partly be genetically determined.

The genetic contribution to obesity has been extensively investigated in genome-wide association studies (GWAS) [9,10,11], which successfully discovered susceptible loci and unveiled mechanisms. However, predicting disease risk from genetic background is complicated by interactions between genetic variants and environmental risk factors. Gene–environment interactions are ubiquitous, and may account for the greater part of disease risk seen across genotypes [12]. With rapid advances in omics technologies and analytic approaches, recent genome-wide analyses have reveled genetics of intermediate phenotypes such as circulating metabolites (metabolomics) and gut microbiome [13,14,15,16,17,18]. Integrating information from studies of metabolomics and gut microbiome [19] will provide new insights into the roles of gene–environment interaction in complex traits including obesity, and contribute to a precision prevention and management of obesity. Here, we highlighted data from recent studies of gene–diet interactions on obesity, and discussed how these findings may inform understanding of more complex architecture of interactions between genes and environment factors in obesity and associated diseases.

## 2. Studying Gene–Environment Interactions

More and more genetic bases of complex metabolic diseases such as obesity and type 2 diabetes have been revealed [9,10,11,20,21], however the genetic variants identified so far only explain a small proportion of heritability of the diseases, suggesting so-called ‘missing’ heritability [22]. For example, the recent GWAS by the Genetic Investigation of Anthropometric Traits (GIANT) consortium identified a total of 97 BMI-associated loci; however, these loci only account for 2.7% of BMI variation [11]. As we and others described previously [1,23,24,25,26], the importance of studying gene–environment interaction has been well recognized, and the missing heritability of obesity could be partly due to interactions between the genetic variations and environmental factors such as lifestyle and dietary factors. In the following section of our review, we showed several studies on lifestyle and dietary factors that magnified risk of obesity among individuals genetically at high risk. Genes can trigger the occurrence of diseases when a person with a high-risk genetic profile is exposed to high-risk environmental factors in the gene–environment interaction phenomena [27]. Gene–environment interaction may reflect a causal mechanism where the variants and environmental exposures contribute to the causation of a disease or condition in the same individual with the genetic factors influencing the sensitivity to environmental factors. How these two exposures synergistically affect vulnerability to diseases remains unresolved. In particular, obese individuals are characterized by different body shapes and considerable heterogeneity within the spectrum of clinical obesity may exist [28,29,30,31,32,33,34,35,36,37]. A recent GWAS identified genetic variants associated with overall body shape based on a combination of multiple anthropometric traits [38]. To support gene–diet interaction and precision nutrition in obesity, considering different body shapes and subtypes of obesity would be necessary.

## 3. Dietary and Lifestyle Factors Interact with Genetic Variants on Obesity

Epidemiological studies have consistently shown that particular diets and lifestyles accentuate risk of obesity among adults genetically at high risk (Table 1). For example, replicable evidence has shown that sugar-sweetened beverages [39,40,41], fried food consumption [42], physical activity and sedentary lifestyles [43,44,45] are interacted with genetic variants in the association of obesity.

Intake of free sugars or sugar sweetened beverages is a determinant of body weight [48,49]. We previously reported significant interactions between genetic factors linked to obesity (as assessed by genetic risk score (GRS) based on 32 BMI-associated loci) and intake of sugar-sweetened beverages in two US cohorts of the Nurses’ Health Study (NHS) and the Health Professionals Follow-up Study (HPFS) [39]. The genetic association with obesity was stronger among individuals with higher intake of sugar-sweetened beverages as compared with those with lower intake [39]. In a recent study of Swedish adults, similar findings were observed, and the association of sugar-sweetened beverages with BMI was stronger in people genetically predisposed to obesity [40]. Further, another recent study reported similar interactions between a GRS for obesity and soft drinks consumption in relation to changes in BMI [41]. In a Hispanic population living in Costa Rica, there were significant interactions between intake of sugar-sweetened beverages and the chromosome 9p21 variant on myocardial infarction, and high consumption of sugar-sweetened beverages strengthen the genetic risk [50]. Consumption of sugar-sweetened beverages has been implicated in driving the epidemic of obesity [51]; recent reproducible evidence from these studies in the US and European populations suggests potential interactions in the relationship.

Higher fried food intake, which increases energy intake, is considered as one of unhealthy dietary factors that influence risks of general and central obesity [52]. We previously reported for the first time that fried food consumption interacted with genetic background in relation to obesity in the NHS and HPFS cohorts, highlighting the importance of reducing fried food consumption among individuals genetically predisposed to obesity [42]. Our study indicated that *FTO* genotype showed the strongest interaction (*P*_interaction_ < 0.001) among all obesity predisposing variants [42]. In addition to fried food, among participants of the Genetics of Lipid Lowering Drugs and Diet Network (GOLDN) and the Multi-Ethnic Study of Atherosclerosis (MESA) population, higher intake of saturated fatty acids was associated with higher BMI among individuals at a genetically high risk of obesity [46]. In a study of three US cohorts, association of the *APOA2* − 265T > C polymorphism and BMI was modified by saturated fat intake [53]. 

On the other hand, results of the UK Biobank study [45] did not show significant interactions between BMI-GRS and fired-food consumption or fizzy drink intake. Analysis, and the definitions of fizzy drink consumption (such as no data on type were available) and fried-food intake (which was indicated by combined the reported intake of fried chicken and fried potato), were different from other studies [39,40,41,42], and habitual intake of these foods are also differ across study populations.

A recent study including data from 18 cohorts of European ancestry investigated whether a composite score representing healthy diet (which was calculated based on self-reported intakes of whole grains, fish, fruits, vegetables, nuts/seeds and red/processed meats, sweets, sugar-sweetened beverages and fried potatoes) modified associations of genetic variants associated with obesity using GRSs based on 32 BMI- and 14 waist–hip ratio (WHR)-associated single nucleotide polymorphisms (SNPs) [54]. Their results suggested that associations between genetic predisposition and obesity traits were stronger among individuals with healthier diet scores [54].

A number of studies investigated a gene–physical-activity interaction on obesity [43,44,45,55,56]. A meta-analysis has shown that physical activity attenuated the influence of *FTO* variants on obesity in adults [55]. Whereas greater leisure time physical activity attenuated the genetic association, a sedentary lifestyle indicated by prolonged TV watching was found to accentuate genetic predisposition to elevated adiposity [43]. In our previous paper, we demonstrated that in both women and men from the NHS and HPFS cohorts, the genetic association with BMI was strengthened with increased hours of TV watching [43]. A recent study of the UK Biobank study also provides similar results, and the effect of genetic risk of obesity on BMI was stronger for people watching at least four hours of TV per day compared with those watching three hours or less [45]. The UK Biobank study also reported that associations of genetic predisposition and measures of adiposity (such as BMI and waist circumference) were modified by a variety of sleep characteristics including sleep duration, chronotype, day napping, shift work, and night-shift work [47]. Their results showed that the association of genetic risk and adiposity was exacerbated by adverse sleeping characteristics [47].

Childhood obesity is a strong risk factor for metabolic abnormalities in later adulthood [57,58]. In line with evidence in adults, studies have shown that *FTO* rs9939609 genotype was associated with childhood obesity [59,60,61,62], and also suggested an association of the *FTO* variant and dietary intake and preference [59,61,63]. We previously reported results of a combined analysis of 16,094 boys and girls from 14 studies [63], and found that BMI-increasing allele of the *FTO* variant was associated with increased total energy intake, but not with protein, carbohydrate, or fat intake. Also, there was a significant interaction between *FTO* variant and dietary protein intake on BMI, showing that lower protein intake attenuated the association between the *FTO* variant and BMI, with no heterogeneity among the studies [63]. A study suggests an interaction between the *FTO* SNP rs9939609 and socioeconomic status on childhood obesity [64], and some other studies reported gene–environment interactions in childhood obesity [53,65,66]. In a population-based longitudinal study in Brazil, Vitamin D status significantly modified *FTO* effects on weight changes in children, suggesting that *FTO* SNP rs9939609 may affect childhood weight gain, and genotype effects were more pronounced among children with insufficient vitamin D levels [67].

## 4. Genetic Variants Modify the Response to Interventions

It has been reported that how genetic variants modifies effect of dietary intake on weight loss among overweight and obese individuals. In participants of the Preventing Overweight Using Novel Dietary Strategies (POUNDS Lost) Trial [68] and the Dietary Intervention Randomized Controlled Trial (DIRECT) [69], we have performed a series of analyses on gene–diet interactions in obesity and metabolic risk factors (Table 2) [70,71,72,73,74,75,76,77,78,79,80,81,82,83,84,85,86,87,88,89,90,91,92,93]. There have been debates about which dietary intervention is more effective in losing body weight. According to a meta-analysis that assessed effectiveness of different popular diets in improving weight loss among overweight and obese individuals [94], significant weight loss was observed with any low-carbohydrate or low-fat diet, and weight loss differences between individual diets were small [94]. On the other hand, our findings have consistently shown that the effect of low-calorie dietary interventions varying macronutrient content differed according to the genetic background including disease susceptibility, metabolic status and preference to foods or nutrients. In addition, considerable inter-individual variation has long been noted in response to dietary interventions, and genetic variations may at least partly account for such inter-individual variance. For example, low-fat dietary intervention was associated with more weight loss among overweight and obese individuals with *IRS1* rs2943641 CC genotype [81]. Another study indicated that overweight and obese individuals carrying the T allele of *PPM1K* rs1440581 might benefit more in weight loss when undertaking a low-carbohydrate diet [87].

In addition, obese individuals are at high risk of progression to type 2 diabetes, and previous GWASs revealed susceptibility loci for type 2 diabetes [95,96,97,98,99,100,101,102,103,104]. We examined associations between weight-loss diets and a GRS for diabetes based on 31 diabetes-associated variants and assessed 2-year changes in markers of insulin resistance and β cell function in the POUNDS Lost trial [72]. We found that the lower GRS was associated with a greater decrease in fasting insulin, HbA1c, and insulin resistance as assessed by HOMA-IR, and a lesser increase in insulin secretion as assessed by HOMA-B, particularly among participants consuming a low-protein diet [72]. We found a significant interaction between the GRS and dietary protein on these outcomes, and the genetic effect was opposite among who consumed a high-protein diet [72]. Furthermore, we previously showed that changes in adiposity and metabolic response to weight loss diets varying macronutrient content were significantly influenced by several other individual genetic variants, such as those relating obesity (*FTO* and *NPY*), and type 2 diabetes (*TCF7L2* and *IRS1*, etc.). Also, a genetic variant in *FGF21* region determining preference to carbohydrate intake was associated with improving obesity in the POUNDS Lost trial [70]. Our series of studies through assessing gene–diet interaction support a concept of ‘precision dietary interventions’ which takes individual variability, determined by genome, metabolome, microbiome, and other makeup, into consideration in designing interventions. Despite further external replications are necessary, accumulating data suggest that one dietary intervention might be more appropriate than others according to individual variability. Personal information such as genotype is useful to predict inter-individual differences in effectiveness of dietary interventions; however, there are concerns whether provision of such personal information may induce adverse effects for individual’s behaviors. According to results of a randomized controlled trial of healthy middle-aged adults [105], as compared to standard lifestyle advice, additional provision of personalized information about genetic risk (of type 2 diabetes) did not affect behaviors (such as physical activity and dietary habit) among the study participants. Also, provision of personal information about risk of type 2 diabetes did not seem to cause anxiety in their study [105].

In addition to dietary interventions, bariatric surgery is also considered to be an effective treatment for patients with severe and complex obesity [106,107,108]. There is a significant genetic contribution to weight loss after Roux-en-Y gastric bypass (RYGB) surgery [109]. However, only a few GWASs were performed previously to identify genetic variants associated with weight-loss response after gastric bypass [110,111], and more work is needed to understand the role of genetics after bariatric surgery. Whether genetic variants may predict the effectiveness of gastric bypass surgery needs to be further examined.

Further, an increasing number of research studies reveal new genetic variants associated with diseases, and whether or how much modifiable factors would alter the genetic risk need to be further investigated in the future. In a recent meta-analysis of diet/lifestyle intervention trials, the effect of weight-loss interventions was not different according to *FTO* risk allele [112]. On the other hand, according to results of the Look AHEAD (Action for Health in Diabetes) trial, genetic risk of coronary artery disease significantly predicted cardiovascular morbidity and mortality over nearly 10 years, and their lifestyle intervention did not alter the genetic association [113].

## 5. Metabolomics Approach in the Gene–Diet Interaction

In addition to classical environmental exposures, circulating metabolites could be used for predicting risk of metabolic diseases [114,115,116] as well as for assessing weight-loss in response to a dietary intervention [91,117]. Metabolomics is systematic study of small-molecules generated by process of metabolisms, and it has made remarkable progress in understanding underlying mechanisms of metabolic diseases and the risk prediction. Among various metabolites, circulating amino acids such as branched-chain and aromatic amino acids concentrations have been consistently associated with metabolic abnormalities like type 2 diabetes and obesity-associated insulin resistance [27,114,118,119]. Also, taurine metabolism disturbance is closely linked to obesity, insulin resistance and diabetes, and we recently reported that effects of diabetes genetic risk (assessed by 31 diabetes-associated variants) on changes in fasting glucose, insulin, and insulin resistance were significantly modified by circulating taurine among overweight and obese participants in the POUNDS Lost trial [92]. We observed that elevated concentrations of taurine were associated with a greater reduction of insulin resistance among individuals with higher genetic risk of diabetes than those with lower genetic risk [92].

Recent GWASs have revealed loci associated with intermediate phenotypes and circulating metabolites [13,14,15], and these variants would be useful to investigate effects of genetic determinant of metabolites on obesity. According to the Mendelian randomization principle, genetic variants can be a better marker than biomarkers in assessing causal inference, and it is less likely to be affected by confounding and reverse causation. We previously examined relations between a genetic variant determining amino acid metabolites and obesity in the POUNDS Lost trial [87]. We identified significant interactions between dietary fat and a genetic variant rs1440581 near *PPM1K* gene region that was associated with branched-chain amino acids/aromatic amino acids ratio (the Fischer’s ratio) on weight loss and changes in insulin resistance [87]. Our results suggested that biological mechanisms underlying associations of metabolites and the outcomes were different across the participants [87]. More studies are warranted to examine metabolomics approaches in the gene–diet interaction to get insights into potential mechanisms.

## 6. Potential Interactions of Diet with Gut Microbiome

Gut microbiota may be a potential factor for the treatment of obesity and related metabolic diseases [120,121], and a study has also shown that obese individuals with lower bacterial richness would have greater weight gain [120]. Potential influences of dietary habit on gut microbiota have also been attracting interests [19,122,123]. Long-term dietary habits would influence in determining composition of gut microbiota [124], suggesting the importance of well-designed study to investigate the interplay of long-term dietary intake and gut microbiota on metabolic disease onset. Circulating levels of a microbial metabolite, trimethylamine *N*-oxide (TMAO), has been associated with an increased risk of cardiovascular diseases and mortality [125,126], and its precursor such as betaine was also associated with cardiovascular diseases and type 2 diabetes [127,128]. While experimental studies in animals suggest the causality of gut microbiota in development of metabolic diseases, prospective cohort studies among healthy individuals are warranted to investigate how altered or changing gut microbiota and their genome (metagenome) are associated with risk of complex diseases.

Recently, several studies identified host genetic variants associated gut microbiota [16,17,18], and a study showed an interaction between host genetics and diet in regulating microbiome composition [16]. The study identified a genome-wide significant variant in *LCT* region that determines gut microbiome *Bifidobacterium* abundance, and the variant was also differently associated with dairy intake [16]. In a study of elderly Mediterranean population, an association of the *LCT* variant and obesity was significantly modified by dairy lactose and milk intake [129], suggesting that changes in the gut microbiota across the *LCT* genotype might be involved in differences in caloric extraction of ingested food and the risk of obesity [129]. Further studies considering gut-microbiome, those related genetic variants, and dietary habit would be warranted.

## 7. Challenges and Opportunities for Gene–Diet Interaction Studies

A major challenge of examining gene–diet interactions is whether observations are replicable in other populations [23,24,25]. In previous publications on gene–diet interactions on obesity, results from different populations are presented to demonstrate that the findings are replicable in other cohorts [39,40,54]. On the other hand, large-scale collaboration studies are also needed to provide a higher level of evidence and also to perform more detailed analyses including different types of dietary factors, phenotypes, and different obesity GRSs. Within the Cohorts for Heart and Aging Research in Genomic Epidemiology (CHARGE) consortium, authors have collected results from multiple cohorts and meta-analyzed results to examine gene–diet interactions [130,131]. Population-wide biobanks have been established in several countries such the UK [132] and China [133]. Research based on the large sample size of biobanks with the electronic health records, available data on habitual dietary intake (such as using food frequency questionnaires), and other health data will significantly contribute to identification of gene–diet interactions on various health outcomes. Also, it is of importance to provide robust evidence on gene–environment interaction from a large-scale collaboration study in participants of randomized clinical trials. Different dietary interventions were introduced in each study, and testing gene–diet interactions is also challenging.

Other challenges include imprecise assessment of environmental exposures, difficulty in defining the causal variants, and devising standardized statistical models to detect interactions in different patterns [23,24,25]. A study [45] introduced a negative control variable to control for residual confounding factors, and also considered effects of ‘heteroscedasticity’ since overweight and obese individuals have a wider variance in BMI than non-overweight individuals, and these differences in BMI may create false positive evidence of interaction.

## 8. Conclusions

The obesity epidemic during the past decades has coincided with a profound shift of unhealthy dietary patterns, a sedentary lifestyle, and physical inactivity. Genetic predisposition to obesity may have interacted with such an obesogenic environment in determining the obesity epidemic. Increasing evidence has shown the potential effects of gene–environment interactions on obesity. Data from dietary intervention trials suggest that changes in adiposity and metabolic response to low-calorie weight-loss diets could be significantly modified by genetic variants, especially those related to obesity, type 2 diabetes, metabolism and food preference. While further external replication and a large-scale analysis would be necessary to confirm these findings, the positive results obtained thus far tend to support precision dietary interventions considering genetic predisposition to diseases, genetic variants determining dietary preference and metabolites, as well as phenotypes and intermediate metabolites. The idea of precision nutrition and dietary intervention is considered as each dietary habit and advice is individually tailored to prevent chronic diseases on the basis of genomic background, habitual food and beverage consumption, nutrient intake (especially those contributing to risks of diseases), and also a person’s metabolomics, microbiome, and other omics profiles. On the other hand, few studies investigate potential roles of metabolomics mechanisms and gut microbiome that may act at the interface of genetic variation and environment in affecting obesity and health. Research integrating data on genes, dietary habits, metabolites and gut-microbiome in investigation of human health would be one of the most exciting areas in precision nutrition in the near future.

## Figures and Tables

**Table 1:** Unfavorable lifestyle and dietary factors that may accelerate risk of obesity among individuals genetically at high risk.

Factors	References
High intake of sugar-sweetened beverages	[39,40,41]
High intake of fried food	[42]
High saturated fatty acids intake	[46]
A sedentary lifestyle (indicated by prolonged TV watching)	[43,45]
Sleep characteristics	[47]
Physically inactive lifestyle	[43,44,45]

**Table 2:** Genetic variant that may alter effect of low-fat/high-carbohydrate and high-protein weight-loss diets on obesity and metabolic risk factors among overweight and obese individuals.

Low-Fat/High-Carbohydrate Diet or High-Fat/Low-Carbohydrate Diet		High- or Low-Protein Diet	
Genetic Variants	Outcomes	Genetic Variants	Outcomes
Diabetes genetic risk score [85]	Glycemic traits	Diabetes genetic risk score [72]	Insulin resitence; Insulin secretion
*IRS1* rs1522813, rs2943641 [81,83]	Insulin resistance; Metabolic syndrome; Body weight;	*DHCR7* rs12785878 [84]	Insulin resitence
*FTO* rs1558902 [93]	Insulin resistance	*FTO* rs9939609, rs1558902 [73,90]	Body composition and fat distribution; Appetite
*GIPR* rs2287019 [80]	Glycemic traits; Insulin resistance		
*CRY2* rs11605924, *MTNR1B* rs10830963 [79]	Energy expenditure		
*TCF7L2* rs12255372 [78]	Body composition		
*PCSK7* rs236918 [71]	Insulin resistance		
*APOA5* rs964184 [88]	Lipid profiles		
*LIPC* rs2070895 [86]	Lipid profiles		
*CETP* rs3764261 [82]	Lipid profiles		
*NPY* rs16147 [89]	Blood pressure		
*PPM1K* rs1440581 [87]	Insulin resistance; body weight		
*FGF21* rs838147 [70]	Body composition		
Adiponectin GRS [75]	Appetite		
